# Optimum Selection of Variable Pitch for Chatter Suppression in Face Milling Operations

**DOI:** 10.3390/ma12010112

**Published:** 2018-12-31

**Authors:** Alex Iglesias, Zoltan Dombovari, German Gonzalez, Jokin Munoa, Gabor Stepan

**Affiliations:** 1 Dynamics and Control, IK4-Ideko, 20870 Elgoibar, Basque Country, Spain; jmunoa@ideko.es; 2 Department of Applied Mechanics, Budapest University of Technology and Economics, H-1521 Budapest, Hungary; dombovari@mm.bme.hu (Z.D.); stepan@mm.bme.hu (G.S.); 3 Karlsruhe Institute of Technology (KIT), wbk Institute of Production Science, Kaiserstr. 12, 76131 Karlsruhe, Germany; ggonzalez@ideko.es

**Keywords:** Milling stability, variable pitch, chatter, self-excitation

## Abstract

Cutting capacity can be seriously limited in heavy duty face milling processes due to self-excited structural vibrations. Special geometry tools and, specifically, variable pitch milling tools have been extensively used in aeronautic applications with the purpose of removing these detrimental chatter vibrations, where high frequency chatter related to slender tools or thin walls limits productivity. However, the application of this technique in heavy duty face milling operations has not been thoroughly explored. In this paper, a method for the definition of the optimum angles between inserts is presented, based on the optimum pitch angle and the stabilizability diagrams. These diagrams are obtained through the brute force (BF) iterative method, which basically consists of an iterative maximization of the stability by using the semidiscretization method. From the observed results, hints for the selection of the optimum pitch pattern and the optimum values of the angles between inserts are presented. A practical application is implemented and the cutting performance when using an optimized variable pitch tool is assessed. It is concluded that with an optimum selection of the pitch, the material removal rate can be improved up to three times. Finally, the existence of two more different stability lobe families related to the saddle-node and flip type stability losses is demonstrated.

## 1. Introduction

The machining of components for the energy sector implies high material removal rates in heavy duty machining processes. Current trends point towards the development of even higher power components in nuclear plants and wind turbine applications, which results in more demanding machining processes and higher cutting capacity requirements in production means, due to the larger component size and, thus, bigger material stocks to remove.

**Figure 1:**
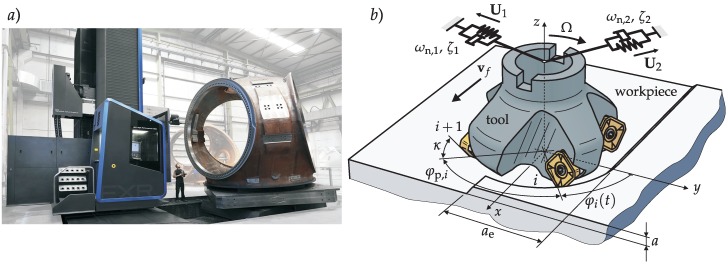
(**a**) Turbine hub machining in a floor-type milling machine with an extensible ram, (**b**) geometry of the considered inserted face milling cutter.

The material range to machine is very broad ranging from the typical steel or cast iron used for wind turbine components to heat-resistant alloys used in the nuclear industry. The big size of the components of the energy sector to manufacture brings along the need of flexible machines. The floor-type milling machine with an extensible ram is one of the most common architectures for this purpose (Figure 1a).

The higher requirements in terms of productivity pave the way for self-excited vibrations, also commonly known as chatter vibrations, which are one of the main challenges linked to heavy duty milling processes. In this case, the chatter vibrations are usually linked to structural low frequency chatter, which are extremely detrimental. Among the adverse effects of this type of vibrations, bad surface finish, excessive tool wear, and even tool or part damage can be mentioned. 

Many works and scientific research have been devoted to chatter avoidance technique development throughout the years [1]. Depending on the source of vibration, which has to be evaluated beforehand, and the stability lobe region the process takes place in, the most appropriate technique can be selected for each specific case [1].

For heavy duty machining operations (Figure 1a), passive [2,3] and active damping [4] of the structure subject to vibrations has been a commonly proposed solution for chatter suppression. Passive dampers usually require big masses and occupied volume due to the high modal masses involved in the critical modes, which usually makes this type of solution unfeasible. Active solutions, on the other hand, are usually rather complex and costly, due to the necessary hardware and controller. Active damping can also be achieved through a machine’s own drives [5], although the effectiveness of this type of technique is very limited due to the long actuation distance from the cutting point and the non-collocated control.

Among the control techniques for chatter avoidance, continuous spindle speed variation [6,7] can be highlighted. However, according to Bediaga et al. [7], this is an effective measure when dealing with chatter occurring at high lobe numbers, which is not the case in heavy duty face milling operations, where low frequency chatter places the process at low lobe numbers.

The spindle speed variation based on a tuning procedure [8] is another useful technique whose key point is to match the milling cutting force intrinsic excitation frequencies with the frequency of the critical mode which is causing chatter. When the dynamics of the cutting system are complex and many modes are involved in stability limitation, the use of the stability lobe diagram (SLD) is useful to identify the stability pockets along the applicable spindle speed range.

However, whereas in materials, such as steel or aluminium, the cutting speed can be broadly varied without a detrimental effect for the tool life, when heat-resistant alloys, such as titanium, Inconel, or stainless steel, are machined, the variation range is very narrow [9]. In some extreme cases, a slight variation of the cutting speed could mean a severe decrease in tool life, therefore, it is very important to adjust the speed to the exact cutting speed recommended by the tool manufacturer. In view of this, the recurring solution of spindle speed tuning lacks applicability for heat-resistant alloy machining. Therefore, instead of acting on the cutting conditions, the selection of the optimum tool is a good alternative solution for chatter removal in heat-resistant alloy face milling processes. 

For regular cutters, the tool selection charts developed by Iglesias et al [10] are a useful method for optimum tool diameter to number of inserts (*D*/*Z*) ratio determination. On the other hand, Budak and Kops [11] demonstrated the cutting stability leap that can be achieved with the use of variable pitch cutters when cutting aeronautic components. The lack of spindle speed variation flexibility mentioned above makes the variable pitch a reasonable solution for vibration problems for heat-resistant alloy machining, since they offer a good value for money.

The variable pitch, which was first proposed by Hahn [12], alters the regenerative phases between the past and the present surface patterns, which has a disruptive effect in the typical regenerative effect of chatter that increases stability. Milling tool manufacturers offer off-the-shelf variable pitch cutters for general purpose milling. Altintas et al [13] demonstrated that a random selection can be far from the optimum achievable pitch, with the possibility of even decreasing the stability if an unlucky decision is made.

For this reason, in order to take advantage of the full potential of variable pitch cutters, it is very important to have a variable pitch selection tool. Slavicek [14] analysed tools with only two alternating delays, and proposed an analytical formula to suppress chatter at a certain spindle speed and chatter frequency. Vanherck [15] upgraded Slavicek’s technique by considering more than two pitch angles on the cutter, and Varterasian [16] included experimental results with randomly distributed pitch angles. After these analytical attempts, Tlusty [17] tried to obtain the best design parameters using time domain milling simulations. 

The formulation of the zeroth order approximation (ZOA) approximation in milling permitted the formulation of faster frequency domain procedures for the optimization of the tool geometry [13]. Based on this approximation, Budak [11] introduced an analytical design methodology based on the phase differences between consecutive constant delays. Its effectiveness was confirmed by an industrial application. In the work of Olgac and Sipahi [18], a unique scheme, namely the cluster treatment of characteristic roots paradigm, was used to investigate the effect of two constant delays on the time-averaged dynamics of milling operation with variable pitch cutters. In interrupted milling operations, however, higher harmonics can have significant strength in the regenerative force, causing discrepancies in time averaging methods [19]. 

The application of time domain based stability models can solve these limitations and they can become an important tool for geometry optimization. Following this approach, a new method based on the semidiscretization stability calculation is proposed [20]. Based on the brute force (BF) iterative method recently developed by Stepan et al [21], a new face milling tool design procedure is deployed and applied to a specific face milling operation. Finally, the advantages of the new method with respect to the traditional optimum pitch estimation method are experimentally demonstrated, as well as the existence of a new double period instability related to the variable pitch pattern repetition.

All these approaches are based in theoretical frequency and time domain stability models, where the regenerative effect is deterministically modelled, and the optimal distribution is obtained analytically or numerically. In the last years, optimization procedures based on experimental studies and multi objective optimization have been proposed for force and vibration reduction and surface roughness improvement [22,23]. In the future, similar procedures could be applied for tool geometry optimization.

In the same way as variable pitch cutters are successfully used in the industry for aeronautic heat-resistant alloy machining, where high frequency chatter related to tools and thin walls limits productivity, this work will evaluate their performance on roughing face milling operations, where they are not typically used nowadays. 

## 2. Stability Lobes for Variable Pitch Cutters

In this section, an overview of the method to predict the performance of variable pitch tools using the general dynamic model of milling is presented. It is important to emphasize that variable pitch cutters are introducing regeneration with multiple constant delays, which must be taken into account in the model development. Depending on the number of pitch angles participating in the optimization, a general optimization scheme can be drawn, although some authors suggest that the optimization of just one angle can already make huge improvement [14].

### 2.1. Stability Model for Variable Pitch Cutters 

The different regenerative delays, *τ_i_*, of a variable pitch tool can be defined with the help of the period of the tool, *T* = 2 π/Ω, and the pitch angles, *φ*_p,*I*_, between consecutive edges as Equation (1):

(1) $$\tau_{i}=\frac{\varphi_{p,i}}{\Omega }\;\mathrm{and}\;\Omega =\frac{2\;\pi \;n}{60\;\frac{s}{\min}},$$

where Ω and *n* are the spindle speed in rad/s and rpm, respectively. 

Unlike regular cutters, the principal period, *T*_p_, of the milling process is not the tooth passing period, *T_Z_* = *T*/*Z*. Depending on the actual configuration, it is determined by the natural number divisor. The equation (2) shows an elegant mathematical way to count different pitch angles of the variable pitch tool and determine its main (principal) period:

(2) $$N=\frac{Z}{N_{\tau }}\;\mathrm{and}\;N_{\tau }=\mathrm{rank}\;{\left[\varphi_{p,(k+l-1)\;\mathrm{mod}\;Z}\right]}_{k,\;l=1}^{Z},$$

as *T*_p_ = *T*/*N*.

To derive the motion and the load of the cutting edges, the relative motion of the tool is considered **x** = col *x*, *y*, *z* (see Figure 1b). The *x* direction coincides with the feed direction. An inserted cutter with a regular lead angle, *κ: = κ_i_*, is posed. In case of variable pitch tools, the stationary part of the feeds, (**f**_i_), is not constant for the different inserts, and it can be approximated for a milling operation by Equation (3) in the *x* direction (**f***_i_* = col(*f_i_*, 0, 0)) using the complete feed per revolution, *f* (m/rev), as:

(3) $$f_{i}=f\frac{\varphi_{p,i}}{2\;\pi }\;\mathrm{and}\;f=v_{f}\frac{2\;\pi }{\Omega }\;(v_{f}=\;|v_{f}|).$$

This chip thickness is also affected by the actual and previous vibrations (Δ**x***_i_*). Therefore, the local chip thickness of the *i*th insert can be derived [11,20] considering the chip thickness direction, **n** (*φ_i_*(*t*)) = col(sin *κ* sin *φ_i_*(*t*), sin *κ* cos *φ_i_*(*t*), − cos *κ*):

(4) $$\begin{array}{l} h_{i}(t)=n^{T}(\varphi_{i}(t))\;(f_{i}+\Delta x_{i}(t))=\\ \;(f_{i}+x(t)-x(t-\tau_{i}))\;\sin\;\kappa \sin\;\varphi_{i}(t)+\\ \;(y(t)-y(t-\tau_{i}))\;\sin\;\kappa \cos\;\varphi_{i}(t)\;-\;(z(t)\;-\;z(t-\tau_{i}))\;\cos\;\kappa , \end{array}$$

The angle which determines the position of the *i*th tooth geometrically (see Figure 1b) is *φ_I_*(*t*) = Ω *t* + $\Sigma_{k=1}^{i-1}$ *φ*_p,*i*_. Assuming (for simplicity) linear cutting force characteristics with edge coefficients, **K**_e_, and cutting coefficients, *K*_c,*t*_ **κ**_c_ = *K*_c,*t*_ col(1, *κ*_c,*r*_, *κ*_c,*a*_) (see Figure 1b and [24,25]), the regenerative cutting force has the form:

(5) $$\begin{array}{l} F(t,x(t),x(t-\tau_{k}))=-\frac{a}{\sin\;\kappa }\sum_{i=1}^{Z}g(\varphi_{i}(t))\;T(\varphi_{i}(t))\;(K_{e}+K_{c,t}\kappa_{c}h_{i}(t))=\\ \;G(t)+\frac{a\;K_{c,t}}{\sin\;\kappa }\sum_{i=1}^{Z}A_{i}(t)(x(t)-x(t-\tau_{i})), \end{array}$$

where the transformation matrix, **T**, transfers cutting forces of each flute to the general tool coordinate system (*xyz*), and the screen function, *g*, considers when the insert is in or out of cutting [20] in the radial engagement, *a*_e_. The regenerative cutting force in Equation (5) depends on the present, **x**(*t*), and the delayed, **x**(*t* − *τ_k_*) (*k* = 1, 2, …, *N_τ_*), positions of the tool. Furthermore, it can be separated into a periodic state independent part, **G**(*t*) = **G**(*t* + *T*_p_), and linear state dependent part with periodic coefficient matrices, **A***_i_* (*t*) = **A***_i_* (*t* + *T*_p_). 

By assuming a general linear nonproportionally damped modal formalism, the cutting force can be projected to the different modes and the system can be analyzed in the space state [26]:

(6) $$\dot{q}(t)-[\lambda_{k}]\;q(t)=U^{T}F(t,x(t),x(t-\tau_{k})),$$

where col(**x**, $\dot{x}$) = col(**U**, **U**[*λ_k_*]) **q**. Theoretically, the dynamics in Equation (6) can be described by the following proto transfer function:

**H**(*λ*) = **U**(*λ***I** − [*λ_k_*])^−1^**U**^T^, (7)

where the mass normalized modal transformation matrix is **U** and *λ_k_* = −*ω*_n,*k*_ *ζ_k_* ± j*ω*_n,*k*_ (1 − *ζ_k_*^2^)^1/2^, with damping ratios, *ζ_k_*, and natural frequencies, *ω*_n,*k*_. 

To determine the asymptotic (linear) stability of the stationary solution, **x**_p_, the variational equation is derived by using perturbation **y** as **x** = **x**_p_ + **y** (**q** = **q**_p_ + **u**), resulting in the following form from Equation (6):

(8) $$\dot{u}(t)-[\lambda_{k}]\;u(t)=U^{T}\left(\frac{a\;K_{c,t}}{\sin\;\kappa }\sum_{i=1}^{Z}A_{i}(t)(y(t)-y(t-\tau_{i}))\right).$$

**Figure 2:**
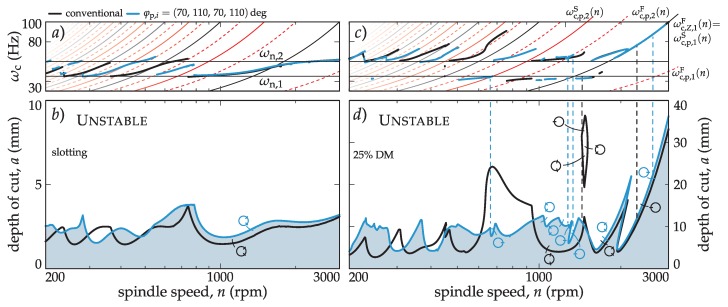
Comparison of milling stability for a four insert (*Z* = 4) regular pitch tool versus a variable pitch tool, *φ*_p,*i*_ = (70, 110, 70, 110) deg in case of slotting (**a,b**) and 25% down milling (DM, **c,d**).

The asymptotic behaviour of this time domain form can be determined directly by the semidiscretization method [27] or a similar time domain based method [28]. In semidiscretization, the aim is to derive the linear map (transition matrix, **Φ**) between the current complete state, **z**_0_ = col*_k_* **u**(*t* − (*k* − 1)Δ*t*), where (*k* = 1, ..., *m*), and the next period, **z**_1_ = col*_k_* **u**(*T*_p_ + *t* − (*k* − 1)Δ*t*), such as **z**_1_ = **Φ z**_0_. The **Φ** is compiled over the principal period by successively solving up to *T*_p_ = *r* Δ*t* with *m* = ⎡*τ*_max_/Δ*t*⎤ (*τ*_max_ = max*_i_ τ_i_*). If all eigenvalues (‘multipliers’, *μ_k_*) of **Φ** have a magnitude less than the unity, the process is stable. The critical multiplier, *μ*_c_, crosses the unit circle in an arbitrary position for a Hopf kind of stability loss, crosses at −1 in the case of flip, while the saddle type of stability loss accompanies *μ*_c_ = 1 (see tiny unit circles in Figure 2).

Alternatively, to semi discretization, the problem can be analysed in the frequency domain [11,13]. According to the Floquet theory, the following trial solution can be assumed and the Fourier expansion of **A***_i_* using Ω_p_ = 2π/*T*_p_ can be performed:

(9) $$y(t)=\sum_{k=-\infty }^{\infty }Y_{k}e^{jk\Omega_{p}t}e^{\lambda t}\;\mathrm{and}\;A_{i}(t)=\sum_{l=-\infty }^{\infty }A_{l,i}e^{j\;l\;\Omega_{p}\;t},$$

Equation (8) can be rewritten in the Fourier domain (*λ* = j*ω*) by using Equation (7) and Equation (9) in the following form:

(10) $$\left(I-\frac{a\;K_{c,t}}{\sin\;\kappa }\Phi \;(\omega )\sum_{i=1}^{Z}\;(I-e^{-j\;\omega \;\tau_{i}}\;E_{i})\;D_{i}\right)\;Y=0,$$

considering *h* modulations of the vibration frequency, *ω*:

(11) $$\begin{array}{c} \Phi (\omega )=\overset{h}{\underset{l=-h}{\mathrm{diag}}}\;H(\omega +l\;\Omega_{p}),\;E_{i}=\overset{h}{\underset{l=-h}{\mathrm{diag}}}\;I\;e^{-j\;l\;\varphi_{p,i}},\\ D_{i}={\left[A_{l-k,i}\right]}_{l,k=\;-\;h}^{h}\;\mathrm{and}\;Y={\mathrm{col}}_{k=\;-\;h}^{h}Y_{k}. \end{array}$$

Non-trivial solution of Equation (10) is granted by:

(12) $$\det\left(I-\frac{a\;K_{c,t}}{\sin\;\kappa }\Phi \;(\omega )\sum_{i=1}^{Z}\;(I-e^{-j\;\omega \;\tau_{i}}\;E_{i})\;D_{i}\right)\;=0,$$

whose solution can be determined in various ways [29,30]. Basically, Equation (12) is a truncated Hill’s representation, which is known as a multi-frequency solution in combination with the frequency response function (FRF) **H**(*ω*):= **H**(j*ω*) Equation (7). The FRF **H**(*ω*), e.g., at the tool tip can be measured experimentally [31], and can be used directly without performing any fitting to determine the modal parameters introduced at Equation (7). In case of variable pitch tools, the different regenerations, *τ_I_*, are summed in an exponential term in Equation (12), while their effects are weighted by **E***_i_* in the modulations. Due to the modulation weight, **E***_i_*, there is no closed form expression of the eigenvalue problem in Equation (12).

However, a zeroth order solution can be introduced by setting *h* = 0 in Equation (12), resulting in the following form:

(13) $$\det\left(I-\frac{a\;K_{c,t}}{\sin\;\kappa }H\;(\omega )\sum_{i=1}^{Z}\;(1-e^{-j\;\omega \;\tau_{i}}\;)\;A_{i,0}\right)\;=0,$$

(14) $$A_{i,0}=-\frac{1}{T_{p}}\int_{0}^{T_{p}}g(\varphi_{i}(t))T(\varphi_{i}(t))\;\kappa_{c}n^{T}(\varphi_{i}(t))dt.$$

The problem with the description of Equation (13) is that there is still no way to deliver an analytical formula as an eigenvalue solution if **A***_i_*_,0_ is under the sum operation. This can be avoided by lifting out common average directional factors, **A**_0_, presented in [32] and defined as:

(15) $$A_{0}:=-\frac{N}{2\;\pi }\int_{\varphi_{\mathrm{en}}}^{\varphi_{\mathrm{ex}}}T(\varphi )\;\kappa_{c}n^{T}(\varphi )\;d\varphi .$$

Thus, it has the form:

(16) $$\det\left(I-\frac{a\;K_{c,t}}{\sin\;\kappa }\sum_{k=1}^{N_{\tau }}\;(1-e^{-j\;\omega \;\tau_{k}})\;H\;(\omega )\;A_{0}\right)\;=0.$$

This single frequency solution [33] is a commonly used method, which offers a good trade-off between accuracy and speed to solve the eigenvalue problem. The angles, *φ*_ex_ and *φ*_en_. in Equation (15) are the exit and entry angles that define the radial immersion, *a*_e_ (Figure 1b).

### 2.2. Stability Property of Variable Pitch Tools

The efficiency of the developed stability model for variable pitch tools has been studied using an example described in the literature [19], where the precision of the different stability models for different cutting engagements was evaluated. It is well known that the effect of the harmonics of the cutting force is not important for large radial immersion (*a*_e_, Figure 1b). However, interrupted cutting with small *a*_e_ boots the periodic nature of the milling process, inducing a period doubling (flip) type of stability loss and mode coupling [19,25]. Since the principal period of the variable pitch tool differs from the tooth passing frequency, a more intricate pattern of period related stability losses are expected. This is demonstrated in Figure 2, where a stability comparison of a regular and a selected variable pitch tool (*φ*_p,*I*_ = (70, 110, 70, 110) deg) for full immersion (slotting) and for 25% down milling (DM) cases is made.

In the slotting case, since in the full immersion case, the periodicity is averaged out for the conventional tool with *Z* = 4, and stays insignificant for the variable pitch case, only the Hopf type stability loss appears. It is clear that the variable pitch can increase the chatter-free depth of the cut only in certain areas. In fact, the introduction of irregular tools can reduce the stability in other spindle speed areas. Therefore, the need for optimization to avoid trial and error is clear.

In the case of interrupted cutting, the previous conclusions are reinforced. Nevertheless, the variable pitch tool shows intricate flip type stability losses on top of the conventional (inherited) stability limits (Figure 2c,d) originating from the different principal periods. 

There are various explanations for the origin of the period doubling instability depending on the different mathematical approaches used for investigating the linear behaviour of the period-one stationary cutting solution (**x**_p_) with the principal period, *T*_p_. From time domain based point of view, the period-one stationary cutting solution of the originally nonlinear system undergoes a period doubling (flip) bifurcation when a period-two orbit emerges with a 2*T*_p_ period [34]. The local behaviour of this period-two orbit can be investigated on a Poincaré section that is normal to the original period-one orbit. On this abstract section, the onset of the period-two orbit appears as an alternating sequence resembling a sequence that flips between two halves of the Poincaré section [35]. 

If some of the regular pitch angles of a milling tool are slightly varied, resulting in *T*_p_ = *T*/*N* (2) principal period, where *N* < *Z*, the original flip instability of a conventional tool with a tooth passing period, *T_Z_* = *T*/*Z*, will be altered. Since *Z* = *N N_τ_* (2), *T*_p_ = *N_τ_ T_Z_* [36], which means the flip critical multiplier, *μ*_c_ = −1, is determined over *T*_p_, resulting in a *μ*_c_ = (−1)*^Nτ^* critical multiplier. Depending on whether *N_τ_* is odd or even, both a *μ*_c_ = −1 and *μ*_c_ = 1 situation can appear. The case, *μ*_c_ = 1, corresponds to a saddle type of stability loss of the corresponding system, which was completely unknown to happen in any milling process. 

Calculating the corresponding vibration frequencies using [37] as:

*ω*_c,*k*_ = *ω*_c,b_ + *l* Ω_p_ = (arg *μ*_c_ + 2π *l*)/*T*_p_, (17)

the modulations, *ω*_c,*l*_, of the chatter frequency, *ω*_c_ (dominant *ω*_c,*l*_), can be determined. In this consideration, it is well known that double period (flip) chatter happens when the chatter frequency, *ω*_c_, and one of its modulated frequencies, *ω*_c,*l*_, with the principal period, *T*_p_ = 2π/Ω_p_, are shaking the same critical mode due to arg *μ*_c_ = ±π (*μ*_c_ = −1). However, keeping the same argument, the saddle kind of stability loss of the stationary solution appears when a modulation is located at zero frequency due to arg *μ*_c_ = 0 (*μ*_c_ = 1), which was never happening in regenerative milling models. This means the dominant chatter frequencies in flip and saddle cases lies on the following lines extracted from Equation (17):

(18) $$\omega_{c,p,l}^{F}(n)=(2l-1)\frac{Z}{N_{\tau }}\frac{n}{120\frac{s}{\min}}(\mathrm{Hz}),\;l\in N,$$

(19) $$\omega_{c,p,l}^{S}(n)=2l\frac{Z}{N_{\tau }}\frac{n}{120\frac{s}{\min}}(\mathrm{Hz}),\;l\in N.$$

In the conventional case, when *N_τ_* = 1, considering that the saddle case instability cannot be present, the flip case can be expressed as:

(20) $$\omega_{c,Z,l}^{F}(n)=(2l-1)Z\frac{n}{120\frac{s}{\min}}(\mathrm{Hz}),\;l\in N.$$

These special lines are presented in Figure 2a,c when *N_τ_* = 2 for the variable pitch case with *φ*_p,*i*_ = (70, 110, 70, 110) deg.

## 3. Variable Pitch Tool Design Process

Several methods to estimate the proper tuning in non-constant pitch tools have been proposed by different authors. In this work, the classical analytical tuning criteria proposed by Slavicek [14] and Budak [32] are briefly introduced and compared with the brute force (BF) iterative method [21], which is based on the semidiscretization stability calculation proposed in the previous section [20,27]. In order to compare different analytical methodologies, altered pitch-deviations are considered, that is, *φ*_p,*i*_ = (*φ*_p_ − Δ*φ*_p_, *φ*_p_ + Δ*φ*_p_, *φ*_p_ − Δ*φ*_p_, …,), when *Z* is even and *N_τ_* = 2 in (2). 

### 3.1. Slavicek´s Methodology

Originally, this method was a graphical way of tuning based on the regenerative phase shifts, *ε*_1_ = *ω τ*_1_= *ω φ*_p,1_/Ω and *ε*_2_ = *ω τ*_2_* = ω φ*_p,2_/Ω, of two subsequent surface undulations based on the scalar single frequency formulation originating from Equation (16) [14]:

(21) $$1-\frac{a\;K_{c,t}}{\sin\;\kappa }\sum_{k=1}^{N_{\tau }}\;(1-e^{-j\;\omega \;\tau_{k}})\;H(\omega )\;A_{0}\;=0.$$

Although this methodology was originally developed for a simple broaching process, it may be also applied for the milling process case by considering a set of technological parameters, where the averaged multi-degrees of freedom, but one dimensional dynamics can describe the process well.

Considering the regenerative effect of the previous surface waves by regenerative phase angles, *ε_i_*, and the dynamics by *H*(*ω*) = *r* (*ω*) e^j *ψ*(*ω*)^, the following expression is obtained [36]:

(22) $$(N_{\tau }-\;(e^{-j\;\varepsilon_{1}(\omega )}+e^{-j\;\varepsilon_{2}(\omega )}))=\frac{\sin\;\kappa }{a\;K_{c,t}\;r(\omega )\;A_{0}}e^{-j\;\psi (\omega )},$$

where the average regenerative phase between two consecutive waves is *ε* = (*ε*_1_ + *ε*_2_)/2 and the phase difference is Δ := Δ*ε*/2 = (*ε*_2_−*ε*_1_)/2 = *ω* Δ*φ*_p_/Ω/2. Using trigonometric relations, the previous equation can be rearranged as:

(23) $$e^{j\;\delta (\omega )}:=(N_{\tau }-\;2\;e^{-j\;\varepsilon (\omega )}\cos\;\Delta (\omega ))=\frac{\sin\;\kappa }{a\;K_{c,t}\;r(\omega )\;A_{0}}e^{-j\;\psi (\omega )}.$$

The resultant phase shift, *δ*, in Equation (23) has the least chance (see [14]) to “intersect” with −*ψ* w.r.t. *ω* if *δ* varies in the slightest way, that is cos Δ ≈ 0. In this case, when the system is less prone to lose stability, an analytical formula can be derived for regenerative phase difference:

(24) $$\Delta =m\frac{\pi }{2},\;\Delta \varphi_{p}=\frac{\Omega }{\omega }(\pi +2k\pi ),$$

where *ω* is the chatter frequency and, e.g., Δ*φ*_p_ = *φ*_p,2_ − *φ*_p,1_ is the pitch difference (*m* = 1, 3, 5, … and *k* = 1, 2, 3, …).

### 3.2. Budak´s Method

This method is based on the single frequency solution explained in detail in [32]. It serves a parametric solution originating from the eigenvalue solution of the multi-dimensional averaged model (16). The expression for the limit depth of the cut for variable pitch tools can be presented as:

(25) $$a(\omega )=-\sin\kappa \frac{2\;\pi }{K_{c,t}}\frac{\Lambda_{I}(\omega )}{S(\omega )},\;\Lambda (\omega )=\Lambda_{R}(\omega )+j\Lambda_{I}(\omega ),$$

where:

(26) $$\Lambda (\omega )=\frac{a}{2\;\pi }\;\frac{K_{c,t}}{\sin\;\kappa }\;\sum_{k=1}^{N_{\tau }}\;(1-e^{-j\;\omega \;\tau_{k}})\;\mathrm{and}\;S(\omega )=\sum_{k=1}^{N_{\tau }}\sin\;\omega \;\tau_{k}.$$

The main concept of the equation (25) is that the depth of the cut will achieve theoretically its maximum when the *S*(*ω*) denominator achieves its minimum, resulting in:

(27) $$\begin{array}{l} S(\omega )=\sin\;\varepsilon_{1}+\sin\;(\varepsilon_{1}+\Delta \varepsilon )\\ \;=2\;\cos\frac{\Delta \varepsilon }{2}\sin\;\frac{\omega }{\Omega }\pi =0\;\Rightarrow \;\cos\frac{\Delta \varepsilon }{2}=0. \end{array}$$

This leads to an equivalent definition to Slavicek´s one. By equating (27), the same expression can be determined as in Equation (24), consequently:

(28) $$\Delta \varphi_{p}=\frac{\Omega }{\omega }(\pi +2k\pi ).$$

Therefore, the criterion for variable pitch tuning is the same as the criterion proposed by Slavicek. Thus, Budak´s and Slavicek´s solutions are considered the same for this case of study, and will be further referred to as the BS tuning method.

### 3.3. BF Methodology

The brute force (BF) iterative method [21,36] was developed to overcome the limitations of previous methodologies. All analytical optimization methods are based on time-averaged models of the milling process. The BF algorithm utilizes the general modelling of semidiscretization, capable of including general dynamics and geometry in process modelling [36].

By using semidiscretization, the magnitude of the Floquet multiplier, *μ* [37], represents the abstract ‘distance’ from the stability boundary (unit circle) in the characteristic complex plane. With this special property of the semidiscretization algorithm, the goal function is to minimize the largest magnitude multiplier, *μ*_max_. To achieve this goal, the BF uses a bisection algorithm on an initial mesh on the design parameters similar to [38]. Possible switches on eigenvalues prevent the use of any gradient algorithm performing efficiently in this kind of parameter optimum search. There are some conditions that can specify the parameter at the optimum regarding the multipliers: (i) The magnitude at the minimum possible value for *μ*_max_ should have a zero derivative with respect to the optimum parameter; (ii) the minimum possible value for *μ*_max_ is set at a parameter point where two multipliers cross. Direct methods are specified separately for (i) and (ii) cases, but they require condition checking, increasing the computational load and the complexity, and losing generality. That is why, to avoid these problems, the robustly convergent bisection algorithm was chosen.

To ease the search for the parameters, the geometry of the regeneration was considered as it was presented first by Comak and Budak [39]. In this case, by using the calculated chatter frequency, *ω*_c_ (17), the regeneration wavelength is determined. The initial mesh is set along that wavelength, avoiding large portions of the largest possible pitch angle span. This in modulo recurrence can be taken into account in the design phase to ensure good chip evacuation for the cutter.

The method works as follows:

1. Selection of technological parameters in the stability chart;
2. perform pre-calculation for the vibration frequency using [37];
3. bisection algorithm is used to iterate the best possible angle; and
4. perform feasibility analysis to check whether the tool can be manufactured at all considering the in modulo recurrences.

### 3.4. Stabilizability (SYD) and Optimal Pitch Angle Diagrams 

**Figure 3:**
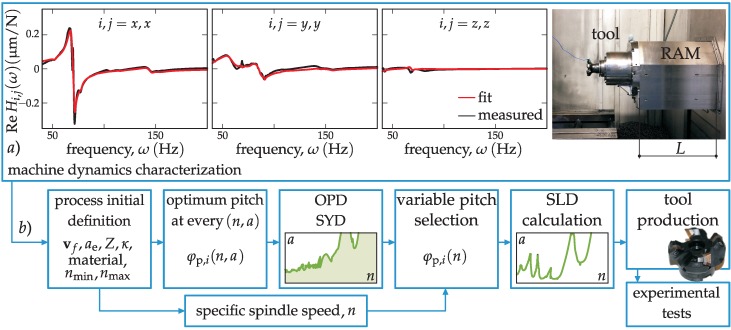
(**a**) Machine dynamics characterization; (**b**) flowchart of the procedure for calculation of the optimal pitch variation.

In this section, the tool selection procedure with the introduction of the stabilizability (SYD) and optimal pitch angle diagram (OPD) is described. All achievable topological cases are investigated based on a real industrial case (Figure 3a). The effect of various topologies is compared and the best possible tool geometry that is capable of improving the process using acceptable resources is derived. Different calculation cases based on the real dynamics of a machine tool (see Figure 3a) are presented for the depicted *L* = 400 mm position, while the process and dynamics details are listed later in the experimental part in Table 1 and Table 2. 

System dynamics and process characteristics must be known beforehand in order to perform the BF method (see Figure 3). Once the optimum pitch distribution is calculated through the BF method, the SYD is built (see Figure 3b) to have a clear picture for choosing the best pitch angle distribution, *φ*_p,*I*_, to gain maximum stability in the depth of cut, *a*. The SYD is the theoretical combined graph, where the optimum pitch distribution is calculated for every spindle speed, *n*, of the (*n*, *a*) plane. Therefore, it shows the hypothetical highest depth of cut *a* one could achieve if the tool pitch is changed speed by speed in (*n*, *a*). Finally, once the final variable pitch is selected, the standard SLD calculation (see Figure 3b) is carried out. This will allow the process planner to perform the final fine tuning of the process, with the possibility of increasing the productivity according to the theoretical simulation and producing the tool physically. Figure 3b explains schematically the steps to follow to determine the proper tuned pitch angle(s).

It must be noted that, in a real industrial case scenario, the optimization is usually carried out for a specific spindle speed, *n*, due to the fact that the spindle speed is mostly given and considered as a hardly changeable technological parameter in the case of hard-to-cut materials [9]. 

### 3.5. The Effect of Different Cutting Tool Topologies

**Figure 4:**
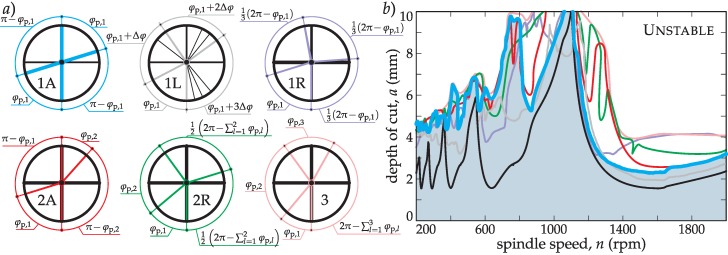
Panel (**a**) presents the different topologies that can be realized easily in a *Z* = 4 milling cutter. Panel (**b**) presents the stabilizability diagram (SYD) for the shown topologies. *φ*_p,min_ = 70 deg.

By introducing the BF tuning methodology, a wide range of different tool topologies are available to perform tuning on multiple pitch angle parameters (see Figure 4a). Multiple parameter optimization is possible by applying a successive search for optimum parameters. This theoretically does not tend to the actual global optimum, but it will practically always be near the optimum. In this manner, multiple angles can be iterated to achieve a higher stability for a certain type of milling process. Naturally, the more parameters are included in the optimization, the more time consuming the optimization is going to be. Thus, it is essential to find the balance between the calculation load and the achievable gain in stability by choosing appropriately the number of optimization parameters.

In Figure 4a, different possible topologies for a *Z* = 4 inserted tool are presented. The main aim is to select the best optimization scenario to tune the pitch distribution of the milling tool in order to increase productivity. It is also important to mention that if more different pitch angles are included in the optimization, this can drastically increase the computation time. Thus, the required time to construct a SYD diagram could be about 100–10,0000 times higher than the time needed to construct simple SLD. 

According to Figure 4b, large improvements can be achieved excluding the first lobe by adopting any kind of optimization procedure. This general improvement means that the simplest alternated topology (1A) combined with BF (BF-1A) optimization should be the most effective one, requiring the least calculation load and providing the highest gain. However, the choice matters in the first lobe (high spindle speed zone, see Figure 4b), where tiny changes in the regenerative phase induce large angular changes in tool design. In this manner, the one parameter optimization with regular angle distribution (BF-1R) shows the largest improvement. 

In summary, in real cases, the optimization of one parameter is enough; it gives the largest gain of stability, which means in most cases, there is no need to perform long multiple parameter optimizations. However, there are areas in the SYD where the inclusion of additional parameters can increase stability (BF-2A, BF-2R in Figure 4b, *n*~1300 rpm). Three parameter optimization serves as an envelope for all other topologies, presenting a minimal gain meaning that it is not worth performing that BF-3 optimization case in an industrial environment. In every calculation, a minimum production bound (*φ*_p,min_ = 70 deg) was kept for the pitch angle to ensure the integrity of the tool and satisfactory chip evacuation grooves. 

### 3.6. Selecting the Optimal Pitch Angle

**Figure 5:**
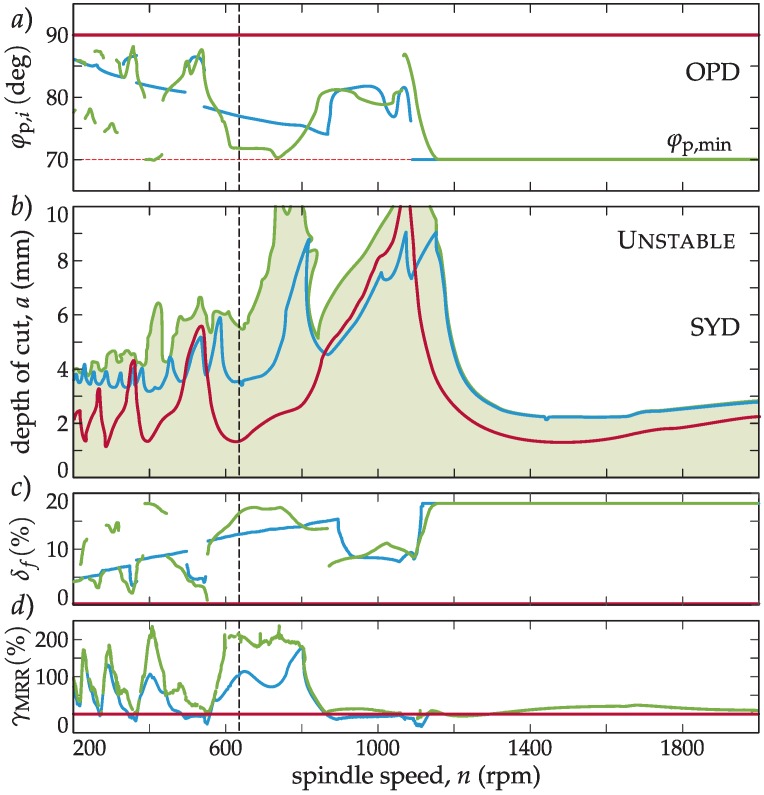
(**a**) Optimal pitch angle chart (OPD), (**b**) stabilizability diagram (SYD), (**c**) relative feed load drop, *δ_f_*, while in (**d**), the relative material removal rate (MRR) gain, *γ*_MRR_, are presented (green: BF-1A, red: regular, blue: BS, and *φ*_p,min_ = 70 deg).

Choosing the 1A topology according to Section 3.5, the optimization of BF and BS algorithms are compared in this section. The optimizations were performed using the industrial case dynamics (Table 1) and process parameters (Table 2) and the resultant OPD (Figure 5a) and SYD (Figure 5b) are determined both for BF and BS algorithms. In both cases, the relative position of the lobes is crucial for the successful application of the variable pitch tool. 

On the one hand, it can be observed that in Figure 5a, at very low spindle speeds (high number of lobes), the optimum angle tends to 90 degrees, because of the smaller and smaller regeneration wavelengths. This means that the required optimum angle variation is so small that it is not feasible to manufacture the pitch variation so accurately. On the other hand, in the first lobes (*k* = 0, 1), the optimum angle, *φ*_p,1_, decreases to really small values (lower than *φ*_p,min_ = 70 deg) and therefore a proper chip evacuation cannot be obtained in some cases. These big variations also unbalance the chip load, and some edges are overloaded (see Equation (3)). 

In the OPD and SYD in Figure 5a,b, it can be observed that the optimum pitch angle distribution selected through the BS method (blue) outperforms the regular pitch tool (red) at practically every spindle speed, whereas the BF-method (green) bests the BS method to a considerable extent.

### 3.7. Productivity of Variable Pitch Tools

Since varying the regular pitch distribution results in larger pitch angle spans, teeth (inserts) will encounter an irregular, but larger feed load per tooth for a given feed speed, *v_f_* (see Figure 1b). However, insert manufacturers define a maximum feed on an insert, *f_Z_*_, max_, which can only be maintained if *f*_max_ is dropped with it as:

(29) $$f_{\max}=\frac{2\;\pi }{\varphi_{p,\;\max}}f_{Z,\;\max}\;\mathrm{and}\;v_{f,\max}=f_{\max}\frac{n}{60\frac{s}{\min}},$$

where *φ*_p,max_ = max*_i_ φ*_p,*i*_. This will directly affect the achievable material removal rate (MRR = *v_f_*_,max_ *a a*_e_). This effect obviously causes a drop in the complete feed per revolution, *f*, however, the pitch variation also causes an increase of stability, $\tilde{a}$, too, which eventually improves MRR.

In this manner, the relative drop on the feed (drop on nominal MRR) can be defined as:

(30) $$\delta_{f}:=\frac{f-f_{\max}}{f}=\frac{Z\;\varphi_{p,\;\max}-2\;\pi }{Z\;\varphi_{p,\;\max}},$$

using the complete original *f = Z f_Z_*_,max_ for the conventional cutter. Figure 5c shows the expected drop in the usable complete feed compared to the conventional tool.

However, if one considers the gain in stability, the relative gain on MRR can be defined as:

(31) $$\gamma_{\mathrm{MRR}}:=\frac{{\mathrm{MRR}}_{\max}-\mathrm{MRR}}{\mathrm{MRR}}=\frac{2\;\pi \tilde{a}-a\;Z\;\varphi_{p,\;\max}}{a\;Z\;\varphi_{p,\;\max}},$$

which clearly shows (Figure 5d) the productivity improvement of these optimized cutters in MRR. With a properly tuned variable pitch tool, around a 200% improvement can be achieved in MRR at certain speeds using the proper tuning, while keeping the chip load predefined by the insert manufacturer and preserving the minimal pitch angle, *φ*_p,min_, to ensure chip evacuation. Thus, productivity is increased and the inserts are not excessively overloaded. 

## 4. Experimental Validation

In this section, the experimental validation of the previous theoretical development is provided. Most of the theoretical calculations were performed in the previous sections using actual data originating from the experimental validation, including Figure 3, Figure 4 and Figure 5. Below, several experimental tests have been carried out, with the following objectives:

1. Show the outperformance of the BF tuned pitch with respect to the constant pitch cutter and the tool selected through BS criterion.

2. Demonstrate that the optimum pitch angle for a particular spindle speed could be the worst if a different spindle speed is used for cutting.

3. Find experimental evidence of the variable pitch pattern related double period instability.

### 4.1. Machine Dynamics Characterization and Process Definition

An industrial case was characterized on a 3-axis Danobat DS630 ram-type machining centre (see Figure 3a), in which a 4-insert (R245-12 T3 K-MM 1040) and 50 mm-diameter tool (Sandvik R245) were used. The machine position was defined at a *L* = 400 mm ram overhang. The dynamic characterization was performed by fitting the experimental FRFs obtained on the tool tip (see Figure 3a) according to [40] and a non-proportionally damped dynamic model was built, which was used for calculation [21]. The used modes are listed in Table 1 by presenting the proportional approximation of modal stiffness, *k*, and mode shapes, **U**.

### 4.2. Objective 1: Optimum Pitch Design Through the BF Method

In this test, the effectiveness of variable pitch tools for chatter suppression is demonstrated, as well as the improvement achieved with the BF criterion with respect to the BS criterion for topology 1A (Figure 4a). For this purpose, the machining of a heat resistant alloy, Jethete M-152, was considered. The technological details of the performed milling process are listed in Table 2.

If the material to cut allowed a wide range of cutting speed variation, the spindle speed could be tuned to the optimum (*n* = 1000–1100 rpm in Figure 5b) and the variable pitch tool would be of no advantage. However, considering the target process of Jethete M152 machining (see Table 2), a particular cutting speed is regarded as optimum. If the spindle speed is shifted from this value, the tool life will decrease dramatically. Moreover, suppliers are not in a position to change any of the main technological parameters, since deviation from the designed one risks the surface integrity of a usually extremely expensive part. The specifically recommended cutting speed for this insert and workpiece material combination was *v*_c_ = 100 m/min, which in this case, corresponded to *n* = 636 rpm. The selected (see Figure 5a,b) and manufactured tools with rounded pitch angles using 1A topology (Figure 4a) for the different tuning methods are shown in Table 3. 

**Figure 6:**
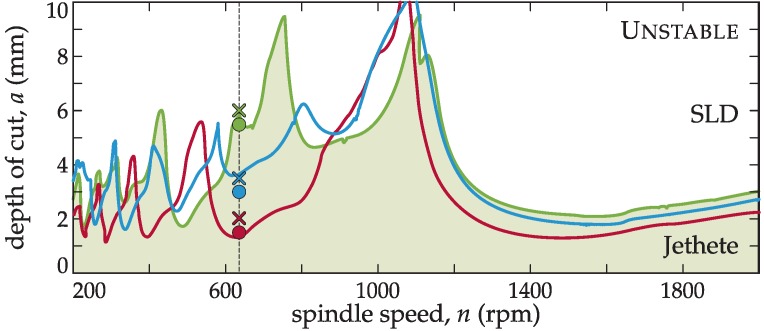
SLD’s and experimental tests for tuned tools (Table 3) for 636 rpm according to Figure 5a,b. The workpiece material is Jethete, restricting the cutting speed to *v*_c_ = 100 m/min (green: BF, red: regular, blue: BS; circle: stable, cross: unstable tests).

Figure 6 shows the predicted ordinary stability diagrams (SLD’s) for the selected angle distributions on a wide frequency range. The depth of cut, *a*, of the process can be adjusted according to the theoretical simulation for the required spindle speed of *n* = 636 rpm. It can be noted that the depth of cut values for the selected spindle speed in the SLD (Figure 6b) were the same as in the SYD in Figure 5b).

The variable pitch tools designed by the presented methods were experimentally tested to assess their cutting performance. The stability of the process was monitored by using industrial accelerometers located close to the cutting point on the three directions as shown in Figure 8b. The experimental cutting results are also plotted on top of Figure 6b, with circles and crosses regarding the stable and unstable tests, respectively.

The stability obtained with the BF tuned tool was three times higher than the constant pitch tool´s stability and more than two times with respect to the tool predicted by the BS method. This tendency was also true for MRR keeping the prescribed maximum chip load per tooth, namely, the BS tuned tool performed with double MRR, while the BF tuned tool three times MRR compared to a conventional inserted cutter (see Figure 5d). With this tool, the maximal removal rate for this spindle speed was obtained, maintaining the recommended cutting speed and a proper surface finish.

### 4.3. Objective 2: Optimum pitch variation with spindle speed

The different methodologies for the variable pitch tuning determine the best pitch for a particular cutting frequency. However, when these non-constant pitch tools are used at a spindle speed different from the tuned one, a severe misbehaviour may occur. To show this stability variation, cutting tests at different speeds were carried out. In this case, a workpiece of C45 steel was used to cover a wide spindle speed range. The target spindle speeds for the stability lobes calculation as shown in Table 4 were *n* = 486, 636, 950, and 1600 rpm.

**Figure 7:**
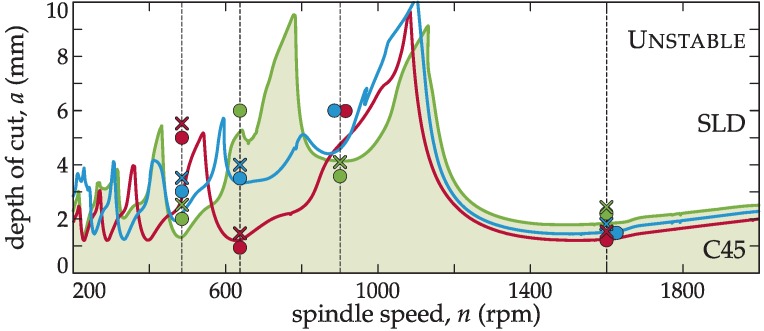
SLD’s of different tuning methodologies for C45 steel workpiece (green: BF, red: regular, blue: BS; circle: stable, cross: unstable tests).

In Figure 7, it is demonstrated that the best tool configuration for a particular spindle speed may produce even lower stability than a constant pitch cutter at a different speed from the target of optimization (see *n* = 950 rpm case in Figure 7).

This means that the classic approach of random selection of variable pitch cutters can even worsen the cutting capability achieved by their regular pitch counterpart.

### 4.4. Objective 3: Experimental Evidence of New Flip Family

For interrupted cutting processes, the apparition of a flip chatter type with constant pitch tools has already been demonstrated [25]. However, the flip instability related to non-constant pitch tools has not been experimentally reported yet. In this test, the existence of a new family of double period or flip bifurcation chatter related to the pitch pattern was proven. In this case, a different position of the machining centre with a longer overhang (*L* = 610 mm) was used for the experimental testing. 

To validate the existence of this new family of double period lobes, an interrupted cutting process was designed, where the tooth passing harmonics were stronger, resulting in dominant and observable flip lobes in the stability diagrams. The process conditions are shown in Table 5, using 10% radial immersion and locating the ram in its more flexible position, *L* = 610 mm, to facilitate the flip domination. With these parameters, new diagrams were calculated and chatter frequencies (Figure 9a) and stability lobes (Figure 9b) were predicted. 

**Figure 8:**
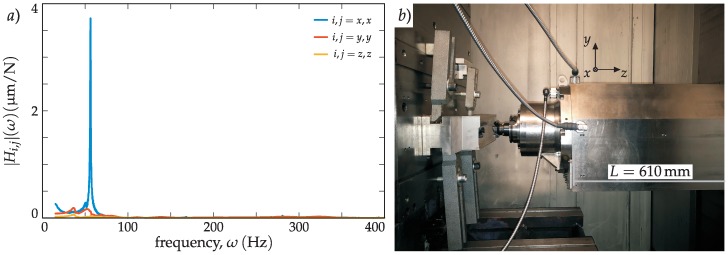
(**a**) shows the direct FRFs with *L* = 610 mm ram overhang presented in (**b**).

In Table 6, the three most important modes are listed, among which the second completely dominated the dynamics (Figure 8a). The fitting was performed for each spatial direction separately, due to the clear dominance of the mode in the *x* direction. 

**Figure 9:**
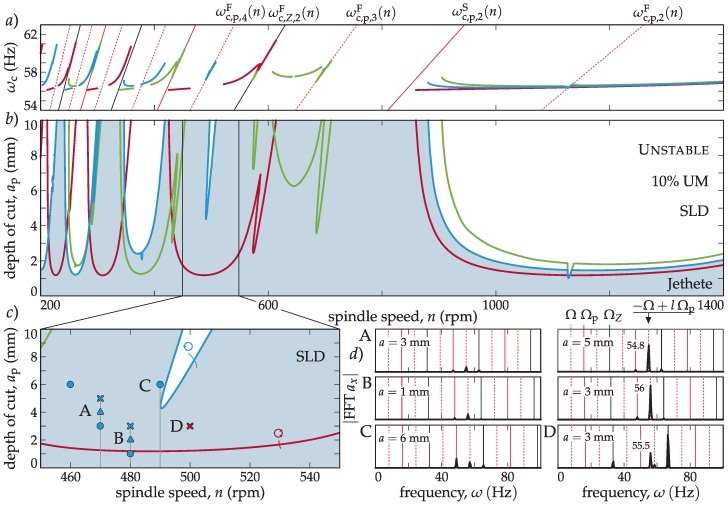
(**a**) presents the chatter frequency diagram, (**b**) shows the SLD’s and the measurements panel (green: BF, red: regular, blue: BS.), while (**c**) is the magnified area where the tests were performed (circle: stable, cross: unstable tests, triangle: marginal). The essential spectra are presented in panel (**d**); a new kind of flip (A, B, C) and Hopf (D) stability losses can be recognized.

The SLD’s (Figure 9b) are presented along with the chatter frequency diagram in Figure 9a, where different flip lobe regions can be observed. The classical flip lobes from a regular cutter corresponding to Equation (20) are lying on the black continuous line in Figure 9a. The new kind of flip lobes corresponding to Equation (18) are lying on the dashed red lines, while the saddle-node type regarding (19) is the continuous red lines shown in Figure 9a. 

Cutting tests were carried out with the three tools selected in Table 3 and the results are shown in Figure 9 for spindle speeds of *n* = 460, 470, 480, 490, and 500 rpm. The existence of the flip lobe related to the variable pitch pattern was shown.

In Figure 9c, the limiting stable and chatter measurement pairs are presented. For the chatter cases, the chatter frequency can be determined by calculating the spectra. These spectra are presented in Figure 9d for the variable pitch BF case in A, B, and C points, and for the regular cutter case, D point. While, in the D point, the dominant chatter frequency was not a harmonic of a spindle speed, referring to a Hopf kind of stability loss; in A and B cases, the dominant chatter frequency was connected to *l* = 4 modulation of *ω*_c,b_ = arg(−1)/*T*_p_ = − π/*T*_p_ = − 2π/*T* = −Ω with Ω_p_ = *N* Ω (*N* = 2 for 1A, (2)) according to Equation (17). This resulted in *ω*_c_ = −Ω + *l* Ω_p_ = 54.8 Hz for point A and *ω*_c_ = −Ω + *l* Ω_p_ = 56 Hz for point B. C was a stable point, which means that the special flip lobe was actually slightly shifted to the lower spindle speed zone. In this manner, the chatter frequency followed the *l* = 4 modulation of the principal frequency, Ω_p_, which is a clear indication of a flip kind of stability loss following the $\omega_{c,p,l}^{F}\left(n\right)$ lines defined in Equation (18).

## 5. Conclusions

The variable pitch cutters can be applied for vibration reduction when a difficult-to-cut material is machined in heavy duty operations limited by structural chatter. The applicability depends on the relative location in the stability lobe. If the chatter problem is lying in the first lobe (*k* < 1), the optimal pitch variations can be too big to assure a proper chip evacuation and can require a reduction in the feed to avoid overloading of some inserts. On the other hand, if it lies in a high number of lobes (*l* > 10), the required pitch variation is so subtle that it can be difficult to practically achieve an optimized performance. The optimal pitch tool chart, stabilizability diagram, and MRR diagrams for variable pitch tools were proposed to consider these limitations and optimize the application of these irregular tools. It was also shown that variable pitch cutters must be used with a moderate feed compared to conventional tools, however, the gain in stability in the axial depth of cut ensured a large improvement in the material removal rate (MRR).

A new variable pitch milling tool design method based on the semidiscretization (SD) and the brute force (BF) method was proposed. An analysis of the different variable pitch patterns for a four insert tool was carried out, concluding that the *φ*_p,*i*_ = (*φ*_p,_ π − *φ*_p_, *φ*_p_, π − *φ*_p_) topology offers a good ratio of performance over the computation time.

The BF method was theoretically and experimentally compared with current state-of-the-art analytical methods, resulting in a clear improvement with respect to those. The BS tuned tool performed with double MRR, while the BF tuned tool three times MRR compared to a conventional inserted cutter

The optimum variable pitch estimation is only valid for a given spindle speed. It was theoretically and experimentally demonstrated that the stability of the variable pitch cutter can be even worse than the one of the regular pitched tool if a different speed from the target of optimization was chosen. Therefore, the tool pitch design procedure developed allows the selection of the right tool, which will help to maximize the MRR for a specific cutting process.

Finally, a new family of double period related stability lobes, which had not been identified in any previous research, was theoretically simulated and experimentally verified. These lobes were produced under the same physical effect as the standard flip lobes. However, in this case, the main harmonics exciting the chatter were those of the variable pitch pattern passing frequency, which originates two more different families of lobes related to saddle and flip type stability losses.

## Figures and Tables

**Table 1:** Modal parameters at *L* = 400 mm ram overhang.

	*ω*_n,*k*_ (Hz)	*ζ_k_* (%)	*Q_k_* (μm/N/s)	~*k_k_* (N/μm)	U*_k_*/|U*_k_*| (1)
1	37.8	4.86	0.09–1.17j	101.36	[0.07 0.75 0.66]^T^
2	58.0	13.27	2.07–3.08j	59.05	[0.22 0.97 0.08]^T^
3	69.6	3.10	1.34–5.30j	41.28	[0.98 0.13 0.11]^T^
4	86.6	5.85	−0.90–2.68j	101.32	[0.09 0.97 0.22]^T^
5	144.8	3.02	−0.48–1.08j	422.24	[0.83 0.55 0.10]^T^

**Table 2:** Process conditions.

*f_Z_* (mm/tooth)	*v*_c_ (m/min)	*n* (rpm)	*a*_e_ (%)	Cutting Direction	*L* (mm)	Workpiece	Cutting Coefficient (N/mm^2^)
0.20	100	636	80	X- up milling	400	Jethete M152	*K*_c, *t*_ = 1843 *K*_c, *r*_ = 625 *K*_c, *a*_ = 467

**Table 3:** Tuned pitch solutions for spindle speed *n* = 636 rpm according to Figure 5a,b).

Tuning Methodology	Pitch Angles, *φ*_p,*i*_ (deg)	Picture
none	(90, 90, 90, 90)	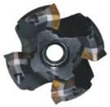
BS	(77, 103, 77, 103)	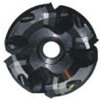
BF	(72, 108, 72, 108)	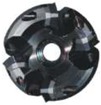

**Table 4:** Process conditions for steel C45 machining.

*f_Z_* (mm/tooth)	*N* (rpm)	*a*_e_ (%)	Cutting Direction	*L* (mm)	Workpiece	Cutting Coefficient (N/mm^2^)
0.20	486 636 950 1600	80	-X Up milling	400	Steel C45	*K*_c, *t*_ = 1836 *K*_c, *r*_ = 734 *K*_c, *a*_ = 387

**Table 5:** Process conditions for new flip lobe family observation.

*f_Z_* (mm/tooth)	*N* (rpm)	*a*_e_ (%)	Cutting Direction	*L* (mm)	Workpiece	Cutting Coefficient (N/mm^2^)
0.20	460 470 480 490 500	10	-X up milling	610	Jethete M152	*K*_c, *t*_ = 1843 *K*_c, *r*_ = 625 *K*_c, *a*_ = 467

**Table 6:** Modal parameters at *L* = 610 mm ram overhang.

*k*	*ω*_n,*k*_ (Hz)	*ζ_k_* (%)	*Q*_k_ (μm/N/s)	*k_k_* (N/μm)	U*_k_*/|U*_k_*| (1)
1	49.8	2.73	0.27–0.59j	264.78	[1 0 0]^T^
2	56.1	0.58	0.66–6.26j	28.19	[1 0 0]^T^
3	53.0	9.90	−0.47–5.24j	31.94	[0 0 1]^T^

## Nomenclature

*a* (m)	axial depth of cut,
*a*_e_ (m)	radial engagement,
**A***_i_* (1)	*i*th periodic directional coefficient matrices in (*x*, *y*, *z*) system,
*g* (1)	screen function of radial immersion,
**G** (N)	only time dependent periodic part of the cutting force,
*h_i_* (m)	local momentary chip thickness on the *i*th edge,
**H** (m/N)	frequency response function (FRF),
*f* (m/rev)	complete feed per revolution,
*f_i_* (m)	feed motion during *i*th delay *τ_i_*,
**f***_i_* (m)	feed vector describing *f_i_* motion in *x* direction,
*f_Z_* (m/tooth)	feed per tooth,
*f_Z_*_,max_ (m)	maximum allowed feed per tooth allowed on an insert,
**F** (N)	momentary resultant cutting force,
*K*_c,*t*_ (N/m^2^)	tangential cutting coefficient,
**K**_e_ (N/m)	edge coefficient vector in (*t*,*r*,*a*) system,
*k_k_* (N/m)	approximated modal stiffness in proportionally damped sense,
*L* (m)	overhang of the RAM of the machine,
*n* (rpm)	spindle speed,
**n** (1)	normal vector to the local edge,
**q** (m (kg/s)^1/2^)	modal displacement vector,
*Q_k_* (m/N/s)	modal scaling factor,
**q**_p_ (m (kg/s)^1/2^)	time periodic stationary solution in modal space,
*r* (m/N)	magnitude of the FRF,
*t* (s)	process time,
(*t*,*r*,*a*)	local edge system,
**T** (1)	transformation matrix from (*t*,*r*,*a*) to (*x*, *y*, *z*) system,
**u** (m (kg/s)^1/2^)	perturbation in modal space for asymptotic analysis,
**U** ((s/kg)^1/2^)	mass normalized modal transformation matrix,
**U***_k_* ((s/kg)^1/2^)	*k*th mass normalized mode shape vector,
(*x*, *y*, *z*)	spatial system,
**x** (m)	spatial displacement vector in spatial directions (*x*, *y*, *z*),
**x**_p_ (m)	spatial time periodic stationary solution,
**y** (m)	spatial perturbation for asymptotic analysis,
*v*_c_ (m/s)	cutting speed,
*v_f_* (m/s)	feed (secondary motion) speed,
MRR (m^3^/s)	material removal rate,
*N* (1)	integer divisor of tool period *T* to the principle period *T*_p_,
*N_τ_* (1)	number of delays,
*T* (s)	tool period,
*T*_p_ (s)	principle period,
*Z* (1)	number of inserts (teeth),
**z** (m (kg/s)^1/2^)	discrete state vector for semidiscretization,
*γ*_MRR_ (1)	relative gain on MRR,
*δ_f_* (1)	relative drop on the complete feed *f*,
*ε* (rad)	regenerative phase,
*ζ_k_* (1)	*k*th damping ratio,
*κ* (rad)	lead angle,
**κ**_c_ (1)	nominal cutting coefficient vector in (*t*,*r*,*a*) system,
*λ* (rad/s)	characteristic exponent,
*λ_k_* (rad/s)	*k*th pole,
*μ* (1)	Floquet multiplier,
*τ_i_* (s)	*i*th delay corresponding to the *i*th pitch angle *φ*_p,*i*_,
*τ*_max_ (s)	maximum delay,
*φ_i_* (rad)	position angle of the *i*th edge,
*φ*_p,*i*_ (rad)	pitch angle between the *i*th and (*i* + 1)th edges,
*ψ* (rad)	phase of the FRF
*ω* (rad/s, Hz)	vibration frequency,
*ω*_c,b_ (rad/s)	base critical (chatter) frequency,
$\omega_{c,p,l}^{F}$ (rad/s, Hz)	*l*th critical frequency of flip stability loss corresponding to the principal period,
$\omega_{c,p,l}^{S}$ (rad/s, Hz)	*l*th critical frequency of the saddle-node stability loss on principal period,
$\omega_{c,Z,l}^{F}$ (rad/s, Hz)	*l*th critical frequency of flip stability loss corresponding to tooth passing period,
*ω*_n,*k*_ (rad/s)	*k*th natural angular frequency,
Δ (rad)	regenerative phase difference,
Δ*t* (s)	time in semidiscretization,
Λ (N/m)	eigenvalue in zeroth order solution,
**Φ** (1)	Floquet transition matrix compiled by semidiscretization,
Ω (rad/s)	angular velocity of the tool,
Ω_p_ (rad/s)	principle angular frequency.
